# Retrospective study of complications following two-stage bilateral total hip arthroplasty: does inter-stage interval matter?

**DOI:** 10.1051/sicotj/2025023

**Published:** 2025-05-22

**Authors:** Camilo Hernán Bonilla-Ortiz, Jorge Eduardo Manrique-González, Andrés Restrepo-Uribe, Juan Manuel Malagón-Santos, Jorge De Francisco Casas-Galindo, Sofia Muñoz-Medina, Jairo Alonso Rincón-Hoyos

**Affiliations:** 1 Orthopaedic Surgeon, Clínica Universitaria Colombia, Fundación Universitaria Sanitas Cra. 66 #23-46 and Cl. 170 #8-41 Bogotá Colombia; 2 Orthopaedic Surgeon, Hip and Knee Specialist, Clínica Universitaria Colombia, Clínica Reina Sofía; Clínica Colsanitas Cra. 66 #23-46 and Av. Cl. 127 #20-78 Bogotá Colombia; 3 MD, MSc, Epidemiology, Unidad de investigación, Fundación Universitaria Sanitas Cl. 170 #8-41 Bogotá Colombia

**Keywords:** Two-stage bilateral total hip arthroplasty, Complications, Transfusion, Thromboembolic events

## Abstract

*Introduction*: This study analyzed complication rates in two-stage bilateral Total Hip Arthroplasty (THA) across three distinct inter-stage intervals to determine the optimal timing for minimizing risk. *Methods*: This was a retrospective, multicentre, analytic study. The three intervals evaluated were <2 weeks (Group A), 2–12 weeks (Group B), and >12 weeks (Group C). The primary outcomes were blood transfusions, thromboembolic events (TVE), and coronary events, and the secondary outcomes were hospital stay, respiratory complications, reintervention, and mortality. The associations between demographic characteristics and complications and the risk hazard of complications were determined. *Results*: A total of 331 patients were included: 86 in Group A, 47 in Group B, and 198 in Group C. Blood transfusions after the second THA were performed in 29.1%, 14.9%, and 7.6% of the time interval groups respectively (*p* = 0.000). One TVE (1.1%) was recorded in group A and 4 (2%) in group C (*p* = 0.613). *Conclusions*: Two-stage bilateral THA with a time interval between both surgeries of <2 weeks presented a significantly higher rate of blood transfusions than longer time intervals between surgeries, with an HR of 2.4 (CI: 95% 1.7–3.3, *p* = 0.000). The incidences of thromboembolic and coronary events were similar between the different timeintervals, demonstrating that two-stage bilateral THA is safe when performed with an interval of at least 2 weeks between both surgeries.

## Introduction

Approximately 42% of patients with hip osteoarthritis may have bilateral involvement [1], of which up to 25% are expected to require total hip replacement [2, 3]. The time interval between the two procedures is controversial [4].

When comparing one-stage bilateral versus unilateral THA, one-stage bilateral THA has shown a significantly higher rate of pulmonary embolism (1.6% vs. 0.7) [5], greater need for transfusion (66% vs. 10%) with an average of 4.3 units of blood transfusion versus 1.1 units, and more extended hospital stay (mean of 14 days vs. 9 days) [6]. The described complications are clinically unfavourable outcomes for patients requiring bilateral hip arthroplasty. Thus, the two-stage procedure is an option for high-risk individuals. Clinical and epidemiological evidence remains inconclusive, and different studies suggest that one-stage bilateral THA can be safe in low-risk patients according to the ASA (American Association of Anesthesiologists) classification [2, 6, 7].

The time interval between both procedures in two-stage bilateral THA should allow homeostasis and tissue repair to enable the patient to tolerate the contralateral hip surgical procedure. However, no standardised evidence-based ideal time interval has been established [2].

This study aimed to identify the incidence of complications occurring at three different time intervals between both surgeries for two-stage bilateral THA to determine the safe timing between procedures.

## Materials and methods

This retrospective, analytical, multicentre study was approved by the Institutional Ethics Committee and was conducted in accordance with the 1964 Helsinki Declaration and its later amendments. Medical records from two institutions were consulted to identify patients aged >18 years who underwent two-stage bilateral THA between January 2009 and June 2018.

Inclusion criteria consisted of patients who underwent primary two-stage bilateral THA due to degenerative or inflammatory pathology and whose records included follow-up information from the first hip operation until 1 year after the second. Subjects were excluded if the medical records data were incomplete. No other exclusion criteria were applied to prevent selection bias. The varied interval times between the two THAs allowed the creation of three groups: (A) patients operated within <2 weeks, (B) from 2 to 12 weeks apart, and (C) >12 weeks apart.

The primary outcomes were blood transfusions, the presence of venous thromboembolism (VTE) events, including deep vein thrombosis (DVT) and pulmonary embolism (PE), and coronary events during the first year. Secondary outcomes included respiratory complications, such as pneumonia or exacerbation of chronic obstructive pulmonary disease (COPD)/bronchitis; cardiovascular complications, such as cardiac failure or arrhythmias; early and late local infections (before or after 6 weeks); early prosthesis dislocation; re-operation; hospital stay; and mortality in the first year.

The time interval between both arthroplasties was determined according to the patient’s physical condition, pain level, and personal preference. Electively and according to the fulfilment of specific criteria from institutional protocols, some patients received outpatient care either for the first, second, or both THAs if they were classified as low-risk ASA, younger than 75 years old, body mass index (BMI) < 30, and absence of cardiorespiratory comorbidities. Antibiotic prophylaxis was applied pre- and post-operatively according to institutional protocol. Cemented, hybrid, or uncement arthroplasty was performed according to international indications and recommendations widely disseminated in the literature [8–10]. Our institutional protocol mandates a minimum preoperative haemoglobin level of 10 g/dL for surgical clearance. Haematocrit and haemoglobin were checked 6 h after completing the procedure. Haemoglobin lower than 8 g/dL or values between 8 and 9 g/dL associated with tachycardia, hypotension, orthostatism, or symptoms of low cardiac output syndrome were considered as transfusion criteria according to institutional protocol.

### Statistical analysis

Qualitative variables were presented as absolute and relative frequencies, whereas quantitative variables were presented through central tendency and dispersion measures. The Shapiro-Wilk test was applied to determine distribution normality. Subsequently, bivariate analysis was performed using the chi-square test or the Mann-Whitney *U* test. Correlations between variables were estimated using the Spearman test. The association between time intervals and the presence of complications was assessed using a Cox proportional hazards model with adjustment for confounding variables including sex, age, ASA, BMI, and comorbidities. Hazard Ratios (HR) were reported with their respective confidence intervals (95% CI). Statistical significance was established at *p* < 0.05.

## Results

During the study period, our four-surgeon hip surgery team utilised a standardised posterolateral approach and surgical technique for all 336 patients who underwent two-stage bilateral primary THA attributed to degenerative etiology. Five patients were excluded due to insufficient information in their medical records. 26.5% of patients had a time interval between both THA of <2 weeks (Group A), 14.2% between 2 and 12 weeks (Group B), and 59.8% of patients had a time interval of >12 weeks (Group C). The demographic and clinical characteristics are summarised in Table 1.

**Table 1:** Demographic and clinical characteristics*.*

Variable	Total	Group A	Group B	Group C	*p*-value
	*N* = 331	*N* = 86	*N* = 47	*N* = 198	
		*n* (26.5%)	*n* (14.2%)	*n* (59.8%)	
Age*	60.2 (13.3)	57.4 (11.4)	58.2 (15.5)	62 (13.3)	0.6
Gender					
Male	93 (28.1%)	30 (34.9%)	10 (21.3%)	53 (26.8%)	0.2
Female	238 (71.9%)	56 (65.1%)	37 (78.7%)	145 (73.2%)	
Weight*	69.1 (12.8)	68.7 (13.5)	66.3 (11.1)	69.9 (12.9)	0.4
Height*	159.9 (8.8)	161.5 (8.8)	157 (9.1)	159.9 (8.6)	0.085
BMI*	27 (4.2)	26.2 (4)	26.9 (4.1)	27.3 (4.3)	0.5
Time interval in weeks#	16.7 (0.42–459)	1.0 (0.4–2)	6.0 (2.4–12)	72.5 (13–459)	0.0001
ASA					
I	41 (12.4%)	14 (16.3%)	9 (19.1%)	18 (9.1%)	0.095
II	239 (72.2%)	64 (74.4%)	32 (68.1%)	143 (72.2%)	
III	42 (12.7%)	5 (5.8%)	6 (12.8%)	31 (15.7%)	
N/I	9 (2.7%)	3 (3.5%)	0 (0.0%)	6 (3.0%)	
Type of prosthesis					
Cemented	96 (29%)	16 (18.6%)	19 (40.4%)	61 (30.8%)	0.0008
Hybrid	152 (45.9%)	36 (41.9%)	23 (48.9%)	93 (47.0%)	
Reverse hybrid	2 (0.6%)	2 (2.3%)	0 (0.0%)	0 (0.0%)	
Uncemented	81 (24.5%)	32 (37.2%)	5 (10.6%)	44 (22.2%)	
Laterality					
Right	150 (45.3%)	39 (45.3%)	18 (38.3%)	93 (47.0%)	0.0135
Left	181 (54.7%)	47 (54.7%)	29 (61.7%)	105 (53.0%)	
Comorbidities					
Rheumatoid arthritis	36 (10.9%)	8 (9.3%)	3 (6.4%)	25 (12.6%)	0.4
VTE	9 (2.7%)	1 (1.2%)	2 (4.3%)	6 (3.0%)	0.5
HTN	127 (38.4%)	20 (23.3%)	23 (49.0%)	84 (42.4%)	0.03
T2DM	32 (9.7%)	3 (3.5%)	4 (8.5%)	25 (12.6%)	0.055
Coronary heart disease	15 (4.5%)	1 (1.2%)	3 (6.4%)	11 (5.6%)	0.2
Heart failure	5 (1.5%)	1 (1.2%)	0 (0.0%)	4 (2.0%)	0.57
Cardiac arrhythmia	12 (3.6%)	1 (1.2%)	3 (6.4%)	8 (4.0%)	0.27
COPD	11 (3.3%)	2 (2.3%)	0 (0.0%)	9 (4.5%)	0.2
Asthma	5 (1.5%)	0 (0.0%)	1 (2.1%)	4 (2.0%)	0.4
Stroke	2 (0.6%)	2 (2.3%)	0 (0.0%)	0 (0.0%)	0.057
Alzheimer	1 (0.3%)	0 (0.0%)	0 (0.0%)	1 (0.5%)	0.7
Dementia	2 (0.6%)	1 (1.2%)	1 (2.1%)	0 (0.0%)	0.18

*Mean and standard deviation (SD); #Mean and ranges. VTE: Venous thromboembolism; HTN: Hypertension; T2DM: Type 2 diabetes mellitus; COPD: Chronic obstructive pulmonary disease.

Hospital stays after the first THA ranged from 0 to 5 days (2 days for 63.1% of patients and 1 day for 21.1%). After the second THA, hospital stays ranged from 0 to 22 days (2 days for 49.2% of patients and 1 day for 35.3%). This data accounts for both inpatient and outpatient procedures. Hospital stays were not significantly different between the first and second THA.

After the first THA, 8.5% of patients required transfusion, while 14.2% needed after the second operation, which was not statistically significant. However, blood transfusion after the second operation was significantly higher in group A than in the other groups, and a tendency to have fewer transfusion requirements was observed with more extended time intervals between both surgeries (Table 2).

**Table 2:** Blood transfusions after the second THA*.*

Variable	Total	Group A	Group B	Group C	*p* value
	*N* = 331	*N* = 86	*N* = 47	*N* = 198	
Blood transfusions	47 (14.2%)	25 (29.1%)	7 (14.9%)	15 (7.6%)	0.000
Mean units transfused	2.3	2.5	1.9	2.2	0.000
Units transfused					
1	3	0	1	2	
2	35	19	6	10	
3	4	3	0	1	
4	6	4	0	2	

The Spearman analysis revealed significant correlations among groups regarding blood transfusion requirement and the number of transfused units*.* No relationship was evident between transfusion and comorbidities, gender, ASA, age, or BMI*.* However, patients with a hybrid prosthesis required more transfusions and units than those with a cemented prosthesis, which was statistically significant (*p* = 0*.*002)*.*

The Cox proportional hazards model showed that group A had a higher risk (HR: 2*.*4; 95% CI: 1*.*7–3*.*3; *p* = <0*.*000) of requiring blood transfusion and needing more units of blood (HR: 1*.*4; 95% CI: 1*.*25–1*.*6; *p* = <0*.*000) than the other two groups*.*

Complications in each group are presented in Table 3*.* Both patients with coronary events had hypertension and were >75 years old*.* One patient presented with cardiac arrhythmia during surgical procedure, and the coronary event occurred 9 months after the second THA*.* The second patient developed a coronary event on the second post-surgical day and was hospitalised for 22 days*.* Both patients had excellent clinical outcomes and survived for a further 1 post-operative year after the second THA*.* Cancer was the cause of death for the two patients who died during the follow-up*.* No significant differences were observed between the groups regarding post-operative complications*.*

**Table 3:** Complications recorded at each surgical procedure time interval*.*

Variable	Total	Group A	Group B	Group C	*p* value
	*N* = 331	*N* = 86	*N* = 47	*N* = 198	
		*n* (26%)	*n* (14.2%)	*n* (59.8%)	
VTE	5 (1.5%)	1 (1.1%)	0 (0.0%)	4 (2.0%)	0.613
DVT	2 (0.6%)	1 (1.1%)	0 (0.0%)	1 (0.5%)	0.413
PE	3 (0.9%)	0 (0.0%)	0 (0.0%)	3 (1.5%)	0.266
Stroke	2 (0.6%)	0 (0.0%)	1 (2.1%)	1 (0.5%)	0.233
Coronary events	2 (0.6%)	0 (0.0%)	0 (0.0%)	2 (1.0%)	0.338
DHF	3 (0.9%)	2 (2.3%)	0 (0.0%)	1 (0.5%)	0.209
Cardiac arrhythmia	1 (0.3%)	0 (0.0%)	0 (0.0%)	1 (0.5%)	0.425
COPD exacerbation/Bronchitis	6 (1.8%)	1 (1.2%)	1 (2.1%)	4 (2.0%)	0.484
Early infection	2 (0.6%)	1 (1.2%)	0 (0.0%)	1 (0.5%)	0.413
Late infection	1 (0.3%)	0 (0.0%)	0 (0.0%)	1 (0.5%)	0.425
Dislocation	15 (4.5%)	5 (5.8%)	1 (2.1%)	9 (4.5%)	0.387
Reintervention	7 (2.1%)	3 (3.5%)	1 (2.1%)	3 (1.5%)	0.367
1st-year mortality	2 (0.6%)	1 (1.2%)	0 (0.0%)	1 (0.5%)	0.413

VTE: Venous thromboembolism; DVT: Deep vein thrombosis; PE: Pulmonary embolism; DHF: Decompensated heart failure; COPD: Chronic obstructive pulmonary disease.

## Discussion

The decision when to perform contralateral operation in two-stage bilateral THA poses a challenge for surgeons. As reported in the literature, the main complications in these cases are thromboembolic events (TVE), cardiovascular complications, and blood transfusions [2, 11] with higher rates observed in patients with ASA 3 and 4 [12, 13]. Regarding transfusions, an incidence of approximately 34% in two-stage bilateral hip replacement with 1-year follow-up have been reported [12].

Our TVE rate was 1.5% (5 cases), and there were no significant differences in TVE cases among the groups. Other authors have published similar rates, with time intervals between surgeries ranging from 1 week to 1 year [12, 14–17].

Only two (1%) coronary events occurred in this study, both in the group of patients with a time interval between surgeries of >12 weeks. Poultsides et al. [15] found no statistically significant differences in acute myocardial infarction between groups of intervals of up to 3 months, 3–6 months, and 6–12 months, with rates of 0.6%, 0.1%, and 0.4% respectively [15]. The low incidence of coronary events suggests that these events are rare and not more frequent in short surgical intervals.

In our cohort, more patients (14.2%) required blood transfusions after the second THA than after the first THA (8.5%), but the difference was not statistically significant. Blood units ranged from 1 to 4 (mean 2.3 units), with two units significantly more frequently transfused. A higher risk of blood transfusion was identified with an HR of 2.4 (CI: 1.7–3.3; *p* = 0.000) in the time interval group of <2 weeks. The transfusion rate in our study appeared to be lower than that previously reported. Parvizi et al. [18] and Taheriazam et al. [19], reported 2.94 units (*p* = 0.001) in the interval of 25 to 303 days [18] and 2.7 units (*p* = 0.541) in the interval of 6 months to 1 year [19]. However, both authors reported cumulative transfusions.

The observed results suggest a higher possibility of blood transfusion after the second THA and a significantly higher need for transfusion in the shortest intervals, which could be explained by haematocrit restoration capacity. Poultsides et al. [15] reported similar results with allogeneic blood transfusions of 24.1%, 21.5%, and 17.7% in intervals of <3 months, 3–6 months, and 6–12 months respectively (*p* < 0.001) [15]. Jie Guo et al. [20] reported a significantly higher rate of cumulative transfusions in patients who underwent the second THA within 30 days of the first compared with longer intervals (*p* < 0.001) [20].

In this study, we did not find any association between blood transfusion and the patient’s comorbidities, sex, age, BMI, or pre-surgical ASA classification; however, a significant association was identified between the replacement type and the rate of transfusion, with hybrid THA duplicating the rate of transfusions compared with cemented THA after the second operation (4.5% vs. 8.5%; *p* = 0.045).

The overall incidence of complications in this study was low, similar to reports from the literature on stage bilateral THA [12, 14, 17]. Based on the low rate of complications observed in this study and according to our experience, even in short time intervals between surgeries such as <2 weeks, the low rate of complications should not represent an obstacle to obtaining satisfactory outcomes in appropriately selected patients with low surgical risk.

The hospital stay durations in this study did not differ between time intervals, favouring the idea that staged bilateral THA with short time intervals between both procedures does not entail higher hospitalisation costs. However, the influence of institutional protocols, including elective outpatient THA, was noteworthy in our cohort, directly affecting hospital stays and costs.

The limitations of our study are its sample size, unequal group sizes, retrospective nature, and potential information bias inherent to the use of medical records as a source of information; however, verification of the primary outcomes of interest was also conducted with other information sources from the medical centres.

To the authors’ knowledge, this is the first study of this kind in Latin America, and it joins the scarce body of literature evaluating and comparing different time intervals between both surgeries in two-stage bilateral THA. It is imperative to optimise the bleeding prevention methods to minimise blood losses that lead to blood transfusions as part of the potential strategies to reduce the risk of complications, particularly for patients whose health status does not allow them to undergo a one-stage bilateral THA.

## Conclusion

Two-stage bilateral THA with a time interval between both surgeries of <2 weeks presents a significantly higher risk of blood transfusions than longer time intervals between surgeries, with an HR of 2.4 (95% CI: 1.7–3.3, *p* = 0.000). The incidences of thromboembolic and coronary events are similar between the different time intervals, demonstrating that two-stage bilateral THA is a safe procedure for the time intervals evaluated in this study.

## Data Availability

The datasets generated during and/or analyzed during the current study are not publicly available for confidentiality reasons but are available from the corresponding author upon reasonable request.
